# Hormonal Regulation of Follicle-Stimulating Hormone Glycosylation in Males

**DOI:** 10.3389/fendo.2019.00017

**Published:** 2019-01-29

**Authors:** Stella Campo, Luz Andreone, Verónica Ambao, Mariela Urrutia, Ricardo S. Calandra, Susana B. Rulli

**Affiliations:** ^1^Centro de Investigaciones Endocrinológicas “Dr. César Bergadá” (CEDIE), Buenos Aires, Argentina; ^2^Instituto de Biología y Medicina Experimental (IBYME-CONICET), Buenos Aires, Argentina

**Keywords:** Follicle-stimulating hormone glycosylation, hormonal regulation, male gonad, Sertoli cell, inhibin

## Abstract

The Follicle-Stimulating Hormone plays an important role in the regulation of gametogenesis. It is synthesized and secreted as a family of glycoforms with differing oligosaccharide structure, biological action, and half-life. The presence of these oligosaccharides is absolutely necessary for the full expression of hormone bioactivity at the level of the target cell. The endocrine milieu modulates the glycosylation of this hormone. During male sexual development a progressive increase in FSH sialylation and in the proportion of glycoforms bearing complex oligosaccharides are the main features in this physiological condition. In late puberty, FSH oligosaccharides are largely processed in the medial- and trans-Golgi cisternae of the gonadotrope and remain without changes throughout adult life. In experimental models, the absence of gonads severely affects FSH sialylation; androgen administration is able to restore the characteristics observed under physiological conditions. The expression of ST6 beta-galactoside alpha-2,6-sialyltransferase 1 is hormonally regulated in the male rat; it decreases after short periods of castration but increases markedly at longer periods of androgen deprivation. Although ST3 beta-galactoside alpha-2,3-sialyltransferase 3 is expressed in the male rat pituitary it is not influenced by changes in the endocrine milieu. The oligosaccharide structure of FSH has an impact on the Sertoli cell endocrine activity. In more advanced stages of Sertoli cell maturation, both sialylation and complexity of the oligosaccharides are involved in the regulation of inhibin B production; moreover, FSH glycoforms bearing incomplete oligosaccharides may enhance the stimulatory effect exerted by gonadal growth factors. In this review, we discuss available information on variation of FSH glycosylation and its hormonal regulation under different physiological and experimental conditions, as well as the effect on Sertoli cell endocrine activity.

## Introduction

Pituitary gonadotropins regulate basic reproductive processes such as gametogenesis, follicular development, and ovulation. Follicle-stimulating hormone (FSH) is synthesized and secreted in multiple molecular forms with different biological characteristics (1–6). Hormone microheterogeneity arises from the post-translational processing of the gonadotropin, which results in molecular variants showing differences in the structure of the oligosaccharides added during glycoprotein biosynthesis (7, 8).

**Figure 1:**
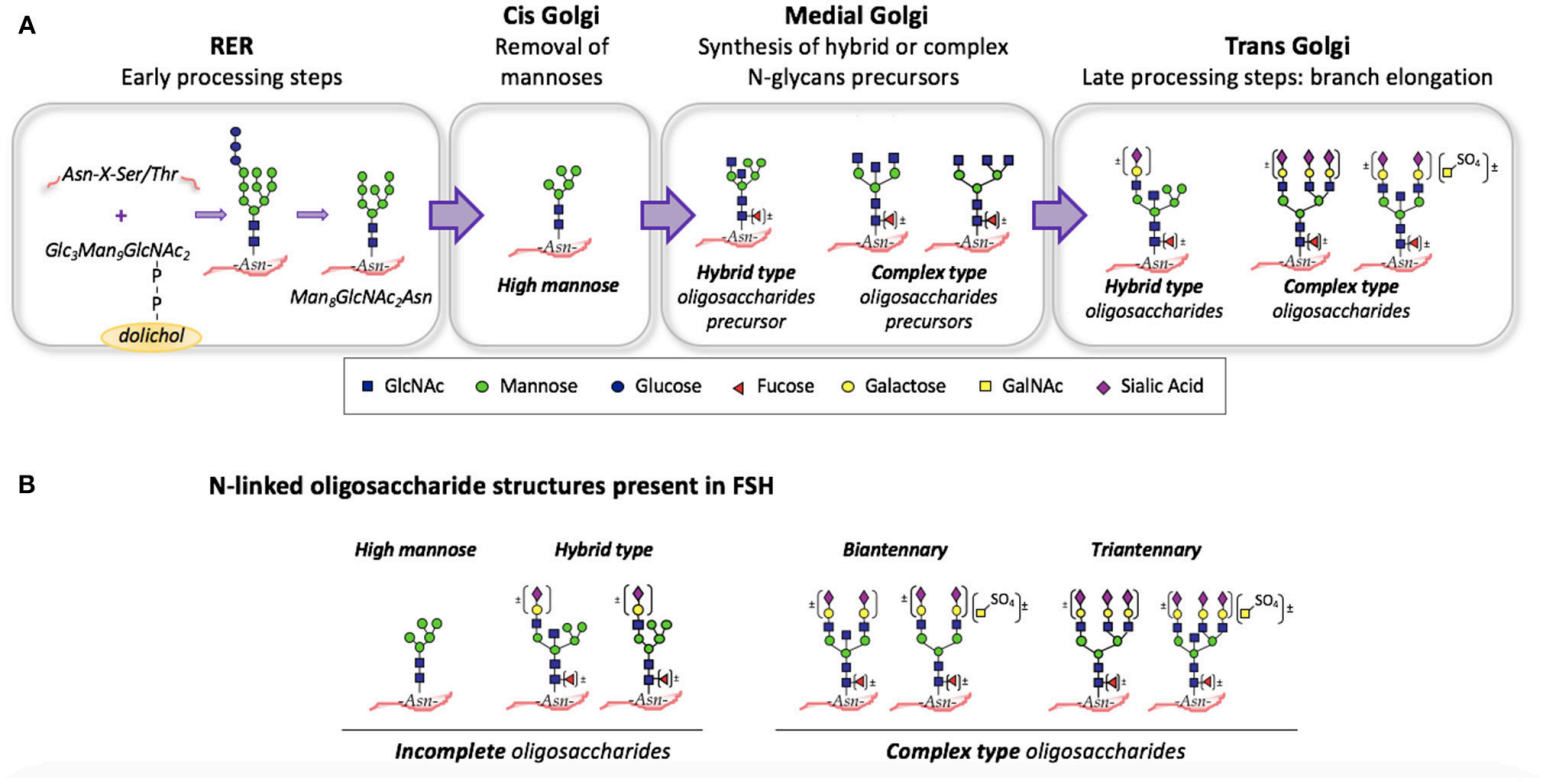
**(A)** Summary of the N-glycan biosynthetic pathway: N-linked glycosylation begins in the RER with the co-translational transfer of a dolichol-linked Glc_3_Man_9_GlcNAc_2_ to a Asn-X-Ser/Thr motif. In the cis-Golgi cisterna, additional mannose residues are removed. In medial-Golgi, hybrid-, and complex-type precursors are formed by the addition of N-acetylglucosamine (GlcNAc) residues. In the trans-Golgi, sequential addition of galactose, and sialic acid occurs. Alternatively, sequential addition of N-acetylgalactosamine (GalNAc) and sulfate (SO_4_) produces sulfated oligosaccharides. **(B)** Some of the N-linked oligosaccharide structures present on human FSH: High mannose and hybrid type N-glycans are incomplete oligosaccharides. Bi- and tri-antennary oligosaccharides are complex type N-glycans. A “bisecting” GlcNAc residue attached to the β-mannose of the core may be present in complex and hybrid-type oligosaccharides. Glycoforms lacking terminal residues such as fucose, galactose, GalNAc, sulfate, and/or sialic acid may also be present.

Gonadotropin glycosylation is a highly complex process; a group of glycosidases (glucosidases and mannosidases) as well as glycosyltransferases (N-acetylglucosaminyltransferases, galactosyltransferases, N-acetylgalactosyltransferases, sialyltransferases, and sulfotransferases) are involved. The initial step in N-linked glycosylation is the co-translational transfer of a dolichol-linked oligosaccharide precursor to specific Asn residues (sequence Asn-X-Ser/Thr) of the nascent polypeptide chain (9–11). When the gonadotropin is still in the rough endoplasmic reticulum (RER), three glucose, and one mannose residues are removed to yield the Man_8_GlcNAc_2_Asn intermediate (Figure 1). Then, glycoproteins are transferred to the Golgi apparatus and removal of additional mannose residues occurs in the cis-Golgi cisterna. This high mannose oligosaccharide serves as substrate for the synthesis of hybrid and complex N-glycans precursors in the medial-Golgi by the addition of N-acetylglucosamine (GlcNAc) residues (9–11). Finally, branch elongation of this precursors occurs in the trans-Golgi; if galactose, and sialic acid are sequentially added, then sialylated oligosaccharides are formed. Alternatively, sequential addition of N-acetylgalactosamine (GalNAc) and sulfate produces sulfated oligosaccharides, which account for almost 10% of human FSH (12, 13) (Figure 1).

Several techniques were used to isolate FSH glycosylation variants with differences in charge, including isoelectric focusing (14, 15), chromatofocusing (16, 17), and zone electrophoresis (18, 19). Likewise, lectin affinity column chromatography was useful to isolate mix of glycoforms with marked differences in the oligosaccharide complexity; these techniques based on the different affinity of sugar residues for a specific lectin maintain the biological activity of the hormone (17, 20–22). Methods based on high-performance liquid chromatography were used to determine glycoprotein oligosaccharide structure. Mass spectrometry allowed to identify carbohydrate composition in digested fragments of a glycoprotein. Matrix-assisted laser desorption ionization (MALDI) and electrospray ionization (ESI) are other optional ionization techniques used for glycan analysis. More recently, the alternative fragmentation technologies of electron capture dissociation (ECD) and electron transfer dissociation (ETD) have been introduced (23). Bousfield et al. (8, 24) have extensively characterized FSH glycoform preparations using nano-electrospray mass spectrometry.

The presence of the oligosaccharides is absolutely necessary for the full expression of FSH bioactivity at the level of the target cell (25–27). The endocrine milieu modulates the glycosylation of this hormone; variations in the relative abundance of FSH glycoforms, with differences in their oligosaccharide structure, have been reported under physiological, and pathological conditions both in males and females (18, 19, 22, 28–31).

Studies carried out in several experimental models have shown the relevance of FSH oligosaccharide structure, particularly sialylation, and oligosaccharide complexity, in the regulation of ovarian function (32). The biological effects of FSH glycosylation variants have been demonstrated for follicular growth, antral formation, and estradiol secretion; furthermore, a specific balance of these glycoforms seems to be required for optimal follicle development (7, 33, 34). Sialylation and complexity of FSH oligosaccharides exert a differential effect on human granulosa cell steroid and peptide production; a less sialylated FSH stimulates the secretion of estradiol, progesterone, free inhibin α-subunit, and inhibin A; whereas more acidic counterparts only affect the production of estradiol and free inhibin α-subunit (35). It has also been shown that the structure of FSH oligosaccharides affects the global gene expression of human granulosa cells (36). The expression of a number of genes involved in regulation of important aspects of granulosa cell function seems to be regulated by FSH carbohydrate structure. In fact, FSH glycosylation variants bearing fully processed carbohydrates modulate the expression of genes associated with biological processes, such as homeostasis, cell differentiation, and apoptosis. The expression of genes related to other essential aspects of granulosa cell function, such as ovarian follicle development, ovulation, response to steroid hormone stimulus, and, in particular, steroid biosynthesis is affected by glycoforms bearing incomplete oligosaccharides.

Pioneering studies carried out by Phillips and Wide (37) and Damian-Matsumura et al. (38) demonstrated that the hormonal milieu regulates the synthesis and secretion of FSH glycosylation variants. The gonadotropin-releasing hormone (GnRH) and sex steroids are recognized endocrine factors involved in the regulation of FSH molecular microheterogeneity, both in females and in males (18, 29, 39, 40). As for the biological relevance of FSH oligosaccharide structure in the regulation of male gonadal function, the available information on variations of FSH glycosylation and its hormonal regulation under different physiological and experimental conditions as well as its effect on endocrine activity in the Sertoli cell are discussed in this review, which includes both experimental and clinical studies.

## Changes in FSH Glycosylation During Male Sexual Development

### Studies in Experimental Models

Early studies carried out in rats (41), lambs (42), and humans (18, 43), demonstrated that FSH oligosaccharide structure and its biological characteristics vary during sexual development in both males and females. Additional evidence showed that the structure of pituitary FSH oligosaccharides changes in terms of complexity and sialylation in immature, prepubertal, and adult male rats (44). FSH glycosylation variants bearing high mannose and hybrid type oligosaccharides are predominant in the pituitaries of immature rats; however, this proportion progressively decreases, and that of glycoforms bearing more complex, highly branched oligosaccharides increases in prepubertal, and adult animals. These variations are closely related to the increase in circulating testosterone levels; thus, a possible androgen influence as well as a hypothalamic contribution through pulsatile GnRH secretion on the FSH glycosylation process may be relevant. Based on these findings and considering that sialic acid can only be added if a galactose residue is already present in the carbohydrate chain, variations in the extent of pituitary FSH sialylation during sexual development in male rats may be expected. The shift toward a more sialylated FSH during sexual development concomitantly with the rise in circulating androgen levels has been previously reported (41, 45). Similar results were obtained by Ambao et al. (15); variations in the distribution profiles of FSH charge analogs were observed at the lower pH intervals of the preparative isoelectrofocusing gradient, where the predominant proportion of FSH was isolated.

### Studies in Humans

Variations in circulating FSH *in vitro* bioactivity and sialylation extent in prepubertal and pubertal normal boys were clearly shown by Phillips et al. (43) and Olivares et al. (46). These authors reported a significant increment in the proportion of more sialylated FSH at the onset of puberty, and they were not able to detect further changes in advanced puberty and adulthood. Not only changes in hormone sialylation but also variations in the oligosaccharide complexity of circulating FSH were described in normal boys during pubertal development (47). A progressive increase in the proportion of FSH glycoforms bearing highly branched oligosaccharides was observed throughout Tanner stages II to IV-V with a concomitant decrease in those FSH glycosylation variants bearing incomplete carbohydrate chains.

Based on these observations it can be deduced that FSH glycosylation in late puberty, in the presence of adult levels of androgens, is characterized by the predominance of glycoforms whose oligosaccharides have been completely processed in the medial- and trans-Golgi cisternae of the gonadotrope.

## Hormonal Regulation of FSH Glycosylation in the Male

Considering the evidence showing variations in FSH molecular microheterogeneity associated with changes in the endocrine milieu, the question arises as whether the hypothalamus, and the testis contribute to the regulation of FSH glycosylation. Hormonal factors, mainly GnRH and androgens, are involved in the regulation of FSH oligosaccharide structure, as demonstrated in rodents and humans (37, 48).

## Studies in Experimental Models

It has been shown that the absence of gonad or the administration of antiandrogens to male golden hamsters and rats increases the proportion of less sialylated FSH in the pituitary gland (40, 49, 50). Studies carried out in the male rat show that castration in prepubertal and adult animals induces changes in the oligosaccharide complexity of pituitary FSH (44). Under these experimental conditions, FSH glycosylation variants bearing incomplete oligosaccharides become predominant as was observed after the administration of the non-steroidal antiandrogen flutamide that blocks androgen action both peripherally and at hypothalamic-pituitary level (51). Interestingly, the proportion of these pituitary FSH glycosylation variants in the absence of androgen action in adults is similar to the one described in the immature male rat. When castrated male rats are treated with dihydrotestosterone, a non-aromatizable androgen, the relative proportion of pituitary FSH glycoforms bearing incomplete oligosaccharides markedly decreases. Concomitantly, a significant increase in FSH glycoforms bearing complex-type oligosaccharides occurs. Thus, androgens are able to restore the characteristic profile of the intact animal. These sex steroids may be needed to regulate the expression of the glycosyltransferases present in the medial Golgi cisternae that determine the FSH oligosaccharide degree of branching.

Not only is the complexity of FSH oligosaccharides altered after castration, but hormone sialylation is also severely affected when testicular function is absent in the male rat. After 4 days of castration a marked decrease in the relative proportion of more sialylated FSH present in the adult pituitary was observed (15). The complete profile of FSH charge analogs shows similar proportions of hormone distributed along the pH gradient of the isoelectrofocusing. Long-term castration further worsens this situation and a considerable amount of hormone is detected at the highest extreme of the pH gradient. The administration of testosterone propionate 2 days after castration is able to restore the physiological distribution profile of pituitary FSH charge analogs characteristic in intact adult animals.

### Studies in Humans

Studying anorchid patients enables us to determine serum FSH glycosylation and the effect of regulatory factors (i.e., GnRH and testosterone) under a condition in which testicular function is absent since early life. Not only are FSH serum levels very high, but also the oligosaccharide structure of the hormone may be altered as well as the response to regulatory factors. In these patients, the oligosaccharide structure of FSH is severely affected and no response to classic regulators factors was observed. The profile of FSH glycosylation variants found in serum of prepubertal and pubertal patients is very different in terms of sialylation and complexity of oligosaccharides to that determined in normal boys (47). There is no difference in the distribution pattern of FSH charge analogs between prepubertal and pubertal anorchid patients; the hormone is distributed in similar proportions throughout the isoelectricfocusing pH gradient. The administration of GnRH to prepubertal anorchid patients for diagnostic purposes does not provoke any change in the characteristics of the charge analogs distribution profile; nevertheless, it induces a discreet increase in FSH serum levels. The classic effect of GnRH described in normal boys is secretion of less sialylated hormone, which does not occur in anorchid patients. Similarly, the administration of testosterone enanthate to pubertal patients to maintain secondary sexual characteristics does not provoke any change in serum FSH levels and does not alter the distribution profile of FSH charge analogs. Based on this evidence, it may be proposed that the addition of terminal sugar residues to FSH carbohydrate branches seems to be a sensitive step in oligosaccharide synthesis in the trans-Golgi cisternae, which may be impaired when a functional gonad is not present during the first years of life.

The distribution profile of FSH glycoforms analyzed in terms of oligosaccharide complexity, either in prepubertal or pubertal patients, does not mimic the one determined in normal boys (47). There is a predominant proportion of FSH bearing biantennary; this characteristic is not observed under physiological conditions at any stage of pubertal development. Nevertheless, after testosterone enanthate administration there is a significant increase in the proportion of FSH bearing complex oligosaccharides. These observations suggest that the glycosyltransferases involved in oligosaccharide branching and in the addition of terminal sugar residues to the carbohydrate chain may have a different response to androgen action.

## Endocrine Regulation of Pituitary Sialyltransferase Expression

Sialic acid content modulates hormone bioactivity; this terminal sugar residue of the oligosaccharide chain determines the half-life of FSH and its metabolic clearance rate (52, 53). On the other hand, more recent studies demonstrated that FSH oligosaccharide structure affects hormone conformation which influences FSHR binding stability and dynamics; thereby, modulates receptor activation and signal transduction (54–57).

It has been described that sialylation of FSH changes under several physiological and pathological conditions concomitantly with variations in the endocrine milieu (18). The question arises as whether the expression of the enzymes that are responsible for sialic acid addition to FSH oligosaccharides may be modulated by endocrine factors. Two sialyltranferases have been identified in the trans-Golgi *cisternae* as responsible for the sialic acid incorporation to FSH carbohydrate chains: ST3 beta-galactoside alpha-2,3-sialyltransferase 3 (ST3GAL3) and ST6 beta-galactoside alpha-2,6-sialyltransferase 1 (ST6GAL1). Damián-Matsumura et al. (38) showed that estradiol modulates mRNA expression of the pituitary *St3gal3* in female rats. The evidence obtained during sexual development and under different experimental conditions in the male rat shows that the pituitary mRNA expression of this enzyme is very low; likewise, very weak staining is observed by immunohistochemistry in tissue sections using a specific antibody (15). Conversely, expression of *St6gal1* is higher than that of *St3gal3* and shows a significant increase in adult rats when compared to immature animals. The effect of castration on *St6gal1* gene expression is intriguing. In the absence of circulating androgens, after 2 days of castration, there is a transient decrease in its mRNA expression. Unexpectedly, at longer periods of castration (5–20 days) there is a progressive increment in the expression of this enzyme. Low mRNA expression for *St3gal3* remains unchanged in all experimental conditions studied. The clear predominance of pituitary FSH glycosylation variants bearing incomplete oligosaccharides after castration may explain the synthesis of less acidic FSH in spite of the presence of the protein and the high expression of *St6gal1* (15). The higher mRNA expression of *St6gal1* than that of *St3gal3* in all the experimental conditions studied further supports the hypothesis that the abundance of FSH charge analogs possessing a 2,6-linked sialic acid is hormonally regulated in male rats.

## Biological Effects of FSH Glycosylation on Sertoli Cell Endocrine Activity

Sertoli cell function mainly depends on the action of FSH; this hormone is responsible for important structural and functional changes that occur during the maturation process. Under the control of FSH, the Sertoli cell secretes estradiol, and inhibins (58, 59). Based on the effect of FSH oligosaccharide structure on steroid and inhibin production by granulosa cells described by Loreti et al. (35), it becomes possible that a similar mechanism may operate in the Sertoli cell. Estradiol production is characteristic of the immature Sertoli cell and it is stimulated by FSH; however, during the maturation process the cell progressively loses the ability to synthesize this steroid (60–62). Creus et al. (17) demonstrated that the FSH sialylation modulates estradiol production in cultured immature rat Sertoli cells. FSH charge analogs isolated at pH higher than five and glycoforms bearing complex oligosaccharides were the most potent stimuli for estradiol production.

**Figure 2:**
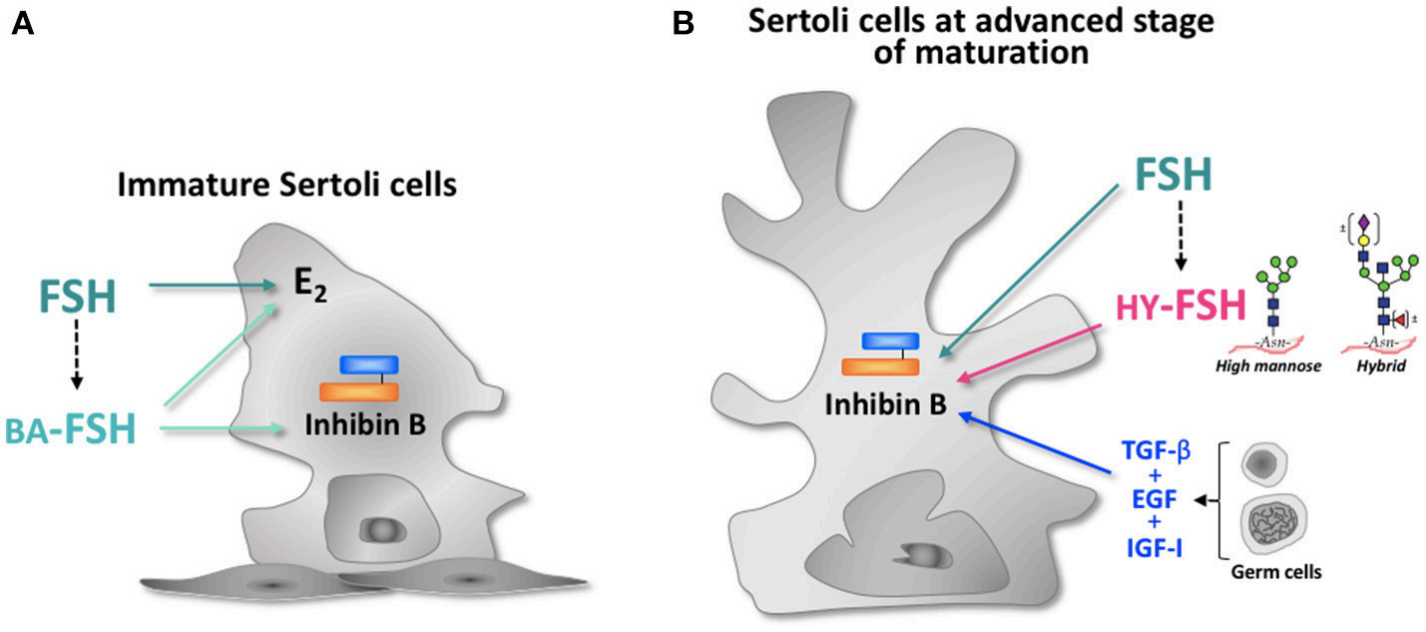
Schematic representation of FSH effect on Sertoli cell endocrine activity at different stages of maturation**. (A)** Immature Sertoli cell: arrows indicate the effects of recombinant human FSH (rhFSH) and basic charge analogs (BA-FSH) isolated at pH 5-7 on estradiol (E_2_) and inhibin B production. **(B)** Sertoli cell at advanced stage of maturation: arrows indicate the effects of rhFSH, growth factors produced by germ cells (TGF-β, EGF, IGF-1), and FSH glycoforms bearing high-mannose and hybrid- type oligosaccharides (HY-FSH) on inhibin B production.

Inhibin B and Anti- Müllerian hormone (AMH) are considered reliable markers of Sertoli cell function in males (63–68). Although production of these two peptides is regulated by FSH, inhibin B serum levels do not always correlate with those of FSH (65, 66, 69). This evidence suggests that different mechanisms are involved in the regulation of both markers. Recombinant human FSH (rhFSH) is unable to increase inhibin B production in cultured immature Sertoli cells, although it stimulates estradiol and cAMP in a dose-dependent manner (70). Interestingly, less sialylated FSH is the only preparation able to further enhance the high amount of inhibin B that these cells produce under basal conditions (Figure 2). As expected, estradiol and cAMP production is markedly stimulated by these charged analogs. Cultured Sertoli cells produce less basal inhibin B than immature cells at advanced stages of maturation (Figure 2) (70). Recombinant human FSH stimulates its production in a dose-dependent manner; nevertheless, growth factors secreted by germ cells are absolutely essential to maintain the synthesis of this dimeric form of inhibin. Interestingly, FSH glycoforms bearing incomplete carbohydrate chains are able to further enhance inhibin B production even in the presence of gonadal factors (70). Based on these findings, it may be proposed that the FSH oligosaccharide structure is involved in the regulatory mechanisms of inhibin production, and interacts with factors produced by testicular cells at different stages of Sertoli cell maturation.

There is strong evidence showing that production of AMH is stimulated by FSH (71–73). Studies in prepubertal FSH-deficient mice showed that treatment with recombinant FSH restores normal serum AMH levels and testicular volume (74). Clinical studies in patients with congenital central hypogonadism and low AMH serum levels, according to their Tanner stage, showed that these parameters were increased after treatment with exogenous FSH (72, 73). The possible participation of FSH oligosaccharide structure in the stimulatory effect exerted by this gonadotropin on AMH production by the Sertoli cell has not been explored yet.

## Concluding Remarks and Future Directions

The regulation of FSH glycosylation seems to be a complex mechanism; GnRH and sex steroids may exert their effect through the expression of different glycosyltransferases involved in the addition and removal of sugar residues. The sialylation of FSH and the complexity of its oligosaccharides affect the biological action of the hormone at the level of the target cell. The presence of a fully functioning gonad seems to be necessary to maintain the secretion of adequate FSH glycosylation variants during sexual maturation and in adult life. Further studies would be necessary to elucidate a possible role of FSH glycosylation on other aspects of Sertoli cell function, including global gene expression, peptide production, and activation of different signal transduction pathways.

The development of new methodologies to improve the isolation of glycoforms without altering the bioactivity of the hormone, would allow to use FSH microheterogeneity as a marker of the hypothalamic- pituitary-gonadal axis function.

## Author Contributions

All authors listed have made a substantial, direct and intellectual contribution to the work, and approved it for publication.

### Conflict of Interest Statement

The authors declare that the research was conducted in the absence of any commercial or financial relationships that could be construed as a potential conflict of interest.
